# Modulation of auditory evoked responses to spectral and temporal changes by behavioral discrimination training

**DOI:** 10.1186/1471-2202-10-143

**Published:** 2009-12-01

**Authors:** Rossitza Draganova, Andreas Wollbrink, Matthias Schulz, Hidehiko Okamoto, Christo Pantev

**Affiliations:** 1 Institute for Biomagnetism and Biosignalanalysis, University of Muenster, Muenster, Germany; 2 Universitätsklinikum Tübingen, MEG Zentrum, Otfried-Müller-Str. 47, D-72076 Tübingen, Germany

## Abstract

**Background:**

Due to auditory experience, musicians have better auditory expertise than non-musicians. An increased neocortical activity during auditory oddball stimulation was observed in different studies for musicians and for non-musicians after discrimination training. This suggests a modification of synaptic strength among simultaneously active neurons due to the training. We used amplitude-modulated tones (AM) presented in an oddball sequence and manipulated their carrier or modulation frequencies. We investigated non-musicians in order to see if behavioral discrimination training could modify the neocortical activity generated by change detection of AM tone attributes (carrier or modulation frequency). Cortical evoked responses like N1 and mismatch negativity (MMN) triggered by sound changes were recorded by a whole head magnetoencephalographic system (MEG). We investigated (i) how the auditory cortex reacts to pitch difference (in carrier frequency) and changes in temporal features (modulation frequency) of AM tones and (ii) how discrimination training modulates the neuronal activity reflecting the transient auditory responses generated in the auditory cortex.

**Results:**

The results showed that, additionally to an improvement of the behavioral discrimination performance, discrimination training of carrier frequency changes significantly modulates the MMN and N1 response amplitudes after the training. This process was accompanied by an attention switch to the deviant stimulus after the training procedure identified by the occurrence of a P3a component. In contrast, the training in discrimination of modulation frequency was not sufficient to improve the behavioral discrimination performance and to alternate the cortical response (MMN) to the modulation frequency change. The N1 amplitude, however, showed significant increase after and one week after the training. Similar to the training in carrier frequency discrimination, a long lasting involuntary attention to the deviant stimulus was observed.

**Conclusion:**

We found that discrimination training differentially modulates the cortical responses to pitch changes and to envelope fluctuation changes of AM tones. This suggests that discrimination between AM tones requires additional neuronal mechanisms compared to discrimination process between pure tones. After the training, the subjects demonstrated an involuntary attention switch to the deviant stimulus (represented by the P3a-component in the MEG) even though attention was not prerequisite.

## Background

Discrimination between sounds coming from the surrounding world is important for survival and for communication in humans and animals. Sound pitch and signal envelope encoding are fundamental for speech and music recognition in humans. Auditory diseases or deficits could impair the discrimination ability for fine differences in frequency or temporal cues in signals leading to communication problems. The improvement of perceptual or functional discrimination ability was investigated in many recent studies. Over the last decades it was clearly shown that plastic changes in the brain, e.g. creation and modulation of synaptic strength between neurons, are not limited to a certain time period. Recent studies in animals, clearly showed that the brain consists of dynamic structures that are remodeled during everyday experience, [1]. Each part of the body (i.e. hand, foot, or tongue, etc.) has a particular spatial representation at the cortex often described in terms of functional maps. The changes that occur in the cortical maps as a result of experience are defined as cortical plasticity. Recanzone et al. [2] trained owl monkeys to achieve fine pitch discrimination in selected frequency ranges. The tonotopic mapping after the discrimination training revealed that the cortical area tuned to the trained frequencies was enlarged by a factor of 2 to 3 compared to untrained monkeys. Bao et al. [3] have shown, that stimulating the ventral tegmental area together with an auditory stimulus of a particular pitch increased its cortical area as well as the selectivity of the neural response to that particular pitch in the primary auditory cortex. This kind of cortical plasticity was discussed in the context of cortical map reorganization, which was based on synaptic plasticity and accounts for perceptual learning [1]. A study of Brown et al. [4], however, concluded that improvement of perceptual discriminative ability could occur without a change in the primary cortex and with only small changes in neuronal response characteristic. In addition, Talvar et al. [5] reported that the cortical reorganization caused by local intracortical micro stimulation of the rat primary auditory cortex did not influence frequency discrimination behavior. A study of Tramo et al. [6] suggested that the auditory cortex is not necessary for normal performance on pure-tone pitch discrimination tasks. However, Ohl et al. [7] reported that between 60% and 95% of the units in the auditory cortex show conditioning-induced changes in the neuronal response by pure tone discrimination training. Dependant on the stimulus conditions, units increase or decrease firing rates in the primary and non-primary auditory cortex, respectively [8-10]. In addition, it was reported that the auditory cortical field in animals includes neurons, which demonstrated experience-induced enhancement and suppression of neuronal responses [11-13]. In general, all these studies suggest that the cortical organization in the auditory cortex is differentially influenced by the neuronal mechanisms involved in encoding and discrimination of different kinds of stimuli. In addition, it can be assumed that the learning mechanisms in the auditory cortex of humans follows similar rules found in animals during comparable tasks. Studies investigating learning-induced plasticity in humans used discriminative task-relevant stimuli, assuming that changes in the synaptic weights (efficacy) between two neurons or in a cell assembly is the substrate of the synaptic or neuronal plasticity leading to amplitude increases of neuronal response to task-relevant stimuli. Enhancement of the magnitude of transient auditory evoked responses (AER) was found in different studies in trained subjects (musicians), compared to non-trained subjects (non-musicians). The N1 and P2 components of the slow evoked response generated in secondary auditory cortical areas are increased in musicians [14-16]. The event-related response that directly reflects discrimination processing in auditory cortex, the so called "Mismatch Negativity" (MMN) described by [17], was also larger in musicians compared to non-musicians, [18].

In an experimental environments small frequencies and envelope (temporal) fluctuations of sounds contributing to speech recognition, can be modeled by amplitude-modulated tones (AM). The analysis of an AM tone starts already in the cochlea along the ascending auditory pathway. The primary auditory cortex (PAC) is responsible for responding to the temporal fluctuation (modulation frequency) of an AM signal [19-21]. These authors found, that the steady-state responses (SSR) were tonotopically represented in the primary auditory cortex depending on the carrier frequencies. Based on this, these stimuli are good candidates for the investigating of discrimination mechanisms in two aspects, spectral (based on changes in the carrier frequency) and temporal (based on changes in the modulation frequency). The discrimination ability between spectral and temporal features after training in monkeys was investigated by varying modulation rates [22,23]. Blake et al. [22] demonstrated that during the learning phase in a frequency discrimination experiment with awake owl monkeys, the response of PAC neurons to the standard tone decreased and responses to the target tones increased. On the other hand, discrimination training between different modulation rates can suppress neuronal activity recorded from PAC neurons by the modulated tone [23]. This clearly shows that training could differently influence the PAC activity depending on the task dependent conditions and type of the stimuli.

The goal of the current study was to investigate the auditory discrimination ability between AM tones differing in spectral (carrier frequencies) or temporal (modulation frequencies) cues by MMN event related response. Taking into account that different neuronal mechanisms are involved in coding of pure tones and coding of AM tones, we expected some deviations from the preliminary results using pure tone discrimination training in humans. Therefore, we investigated possible alterations of the evoked responses to change detection in AM tones in the context of spectral and temporal signal features. Further, the short and long-term training effects on the MMN evoked response generated to stimuli changes (in spectral and temporal aspects) after discrimination training were investigated in order to elucidate different neuronal mechanisms involved in the discrimination learning. Finally, we hypothesized that higher order processes created by the training like involuntary attention to the target stimuli could play a role in this process.

## Methods

### Test subjects

Eleven right-handed subjects (5 females) aged between 22 and 30 years took part in this study. None of these test subjects had a history of otological or neurological disorders. Normal audiological status, defined as an air conduction hearing level threshold of less than 10 dB in the frequency range between 250 and 4000 Hz, was verified by means of pure tone audiometry. Participants gave written informed consent to participation in the study in accordance with procedures approved by the Ethics Commission of the University of Muenster.

### Experimental design

#### Common experimental design

**Figure 1:**
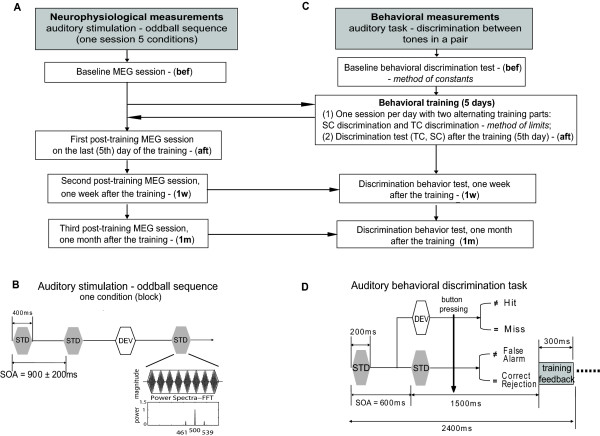
**Experimental block diagram**. (A) Experimental design of the MEG measurements; (B) Stimulus paradigm (oddball sequence) of one condition block in the MEG session, time-series of the standard tone (AM-tone, Fc = 500 Hz, Fm = 39 Hz) and its power spectrum; (C) Experimental design of the behavior measurements; (D) Stimulus paradigm (sequence of tone-pairs of standard-standard or standard-deviant) used in the behavior discrimination training (with feedback) and in discrimination test (without feedback). The MEG and behavioral measurements were performed separately.

The study was composed of two separate parts, neurophysiological and behavioral measurements as illustrated in the graph in Figure 1AC. The acoustic-stimuli paradigms are demonstrated in Figure 1B for the MEG measurements and in Figure 1D for the behavioral measurements.

For all participants a baseline MEG session was carried out prior to the first behavioral discrimination test (*bef*) Figure 1AC. Thereafter they carried out training sessions on five consecutive days (from Monday to Friday). Immediately after the last training session, a second behavioral discrimination test and MEG session were performed (*aft*). One week (*1 w*) and one month (*1 m*) after the training a third and fourth discrimination tests and MEG sessions were carried out, as illustrated in Figure 1AC.

#### Stimuli

The stimuli were amplitude modulated (AM) sinusoidal tones (duration 400 ms, rise and decay time 12.8 ms, 100% modulation depth). An AM tone with a carrier frequency (Fc) of 500 Hz and a modulation frequency (Fm) of 39 Hz was used as a standard stimulus in all sessions. The wave-shape and the power-spectrum of the standard tone are illustrated in Figure 1B. Its power spectrum is characterized by a peak at Fc and two side-band frequencies (Fc-Fm and Fc+Fm), Figure 1B. An AM tone is perceived as pitch at the carrier frequency fluctuating at the modulation frequency. Fluctuations at modulation frequencies around 40 Hz elicit a perception of roughness. The corresponding cortical steady-state response (SSR) to an AM tone is generated at the modulation frequency and is recorded from the primary auditory cortex [24]. Such a tone elicits also a transient response recorded from the non-primary auditory cortex [25].

#### Acoustical paradigm in MEG measurements

The stimuli were presented as standards randomly intermixed with deviants in an oddball sequence (probability of the deviants 20%) as demonstrated in Figure 1B. The sound intensity of all stimuli was equalized to 60 dB above individual sensation level. The inter-stimulus interval from onset to onset, the so-called sound onset asynchrony (SOA), was randomly assigned from 900, 1000 and 1100 ms.

Each MEG session contained two spectral conditions (SC) and three temporal conditions (TC). They were performed in random order in five separate blocks. In one block, we presented only one deviant as shown in Figure 1B, which belonged to either the SC or the TC. In the SC conditions two deviants tone deviate from the standard in carrier frequencies (515 Hz (SC15) and 525 Hz (SC25)), whereas the modulation frequency remains identical for the standard and deviant tones. In order to investigate the effects caused by different modulation rates, three TC conditions were designed. Thereby, deviant tones had a fixed carrier frequency of 500 Hz and deviated from the standard in modulation frequencies by 8 Hz (TC8), 14 Hz (TC14) and 18 Hz (TC18).

#### Acoustical paradigm for the Discrimination Training

To perform the behavioral training and the corresponding discrimination tests a software package developed especially for this purpose (based on **Presentation - *Neurobehavioral Systems Inc***. and **MATLAB**) was used, running on a standard personal computer.

Subjects performed discrimination training for 5 days, preceded and followed by a behavioral discrimination test. The discrimination training was separated in two different blocks (spectral and temporal conditions) performed in one session. Both blocks were randomly presented across the days. Each training block lasted half an hour. Between both training blocks, the subject had a break of 10 min. The training block for each condition was limited to 300 tone pairs, presented binaurally. Standard-Standard (S-S) pairs presented with a probability of 70% contained two AM standard tones with a duration of 200 ms and a 600 ms interval between stimulus onsets, as shown in Figure 1D. The Standard-Deviant combination (S-D) consisting of one standard and one deviant tone was presented with a probability of 30%, the interval between two trains was 2400 ms, Figure 1D. Ten pairs were presented in the beginning of each training block as a pre-test to adapt the subjects to the procedure and were excluded from further analyze. In each training block, five S-S pairs appeared prior to the first S-D pair. The subjects were instructed to concentrate on perceivable differences between tones in a pair, starting with a carrier frequency difference of 25 Hz in the SC and a modulation frequency difference of 74 Hz in the TC. They were asked to press with the right hand the right mouse button when they perceived a difference and the left button, when they did not notice a difference. A stair-case two-alternative, forced-choice procedure with two-down, one-up adaptive rule was used to adjust the deviant stimuli during the training procedure [26]. The adjustment of the deviants followed the rules of the psychophysical method of limits for discrimination threshold detection [16,27,28]. The deviant frequency was changed exponentially dependent on the correct detection (difference decrease) or incorrect response (difference increase) according to the formula:

where **S **was the standard carrier or modulation frequency in SC or TC, respectively, *Δf *is the (carrier or modulation) frequency difference between standard and deviant, *Δf*_0 _is the preceding difference and *f *is a factor, which determines the step width of the learning curve. In this test, *f *was adopted as 0.05. The smallest frequency difference in S-D pairs was 0.96 Hz for the SC and 1.97 Hz for the TC. Within the training blocks the subjects obtained information about the correctness of their responses. They received visual feedback on a monitor situated in front of them (green square - right, red square - wrong), which appeared after pressing the mouse button for 300 ms, Figure 1D.

Four different responses were recorded in the behavioral data analysis. For the S-D pair, a ***Hit ***was registered when a difference between S-D tones was recognized and a ***Miss ***for a response by mistake. Correspondingly, for the pair S-S a ***Correct Rejection ***was collected when the S-S pair was correctly recognized, for a wrong response a ***False alarm ***was registered (Figure 1D).

#### Acoustical paradigm of the behavioral Discrimination test

The discrimination test was performed once before the training session, then immediately after the training and in each post-training phase (after one week and after one month), as shown in Figure 1C. During the behavioral discrimination test, the presentation of the stimuli was the same as in the discrimination training, but without visual feedback. For each condition, 200 stimulus pairs were presented. The S-D pairs were randomly intermixed with 50% probability with the S-S pairs. The carrier frequency difference in the SC was either 2, 3, 5, 10, 15, 20, 25, 30, 40, or 50 Hz. The modulation frequency difference in the TC was set either at 2, 4, 6, 8, 14, 18, 22, 27, 32, or 37 Hz.

The subject's responses (*Hit, Miss, Correct rejection and False alarm*) were collected after one correct or wrong mouse button press. Each discrimination block (SC or TC), contained 10 S-D pairs, was presented randomly in the oddball sequence without adjustment of the deviant tones (method of constants) [27,28].

### MEG Data Acquisition

The MEG recording was performed in a quiet magnetically shielded room using a 275-channel whole-head neuromagnetometer system (Omega2005, VSM-Medtech, Port Coquitlam, BC, Canada). The magnetic field data were sampled at a rate of 300 Hz after low-pass filtering at 100 Hz. The subjects were seated comfortably in an upright position, watching a soundless movie.

### Data Analysis

#### Analysis of Behavioral Data

In the training session, behavioral performance *(P(H) = Hits *divided by the number of the stimulus presentations) was calculated for each frequency of the deviant tone in a S-D pair. The discrimination threshold corresponding to *P(H) = 0.5 *for each day was calculated and plotted on a graph, whereby the x-axis represents the corresponding day of the training session and the y-axis the threshold in Hz [27].

The same calculations were done for the discrimination test, here the discrimination threshold was derived for *P = 0.75*. For this type of test we used a more sensitive method for discrimination and the behavioral performance was calculated according to the formula: *P = [P(H)-P(FA)]/[1-P(FA)]*, where *P(FA) *stands for the probability of the False alarm response. The formula was adopted from [16].

#### Data analysis of MEG Data

The MEG analysis was performed with the CTF Sofware Package. Stimulus related magnetic field data to deviant and standard tones including pre- and post-stimulus intervals (-100 ms to 600 ms, respectively) were collected after rejecting artifact-contaminated epochs in which magnetic field changes larger than 3pT occurred. For each sub-condition (Spectral or Temporal) approximately 100 deviants and 400 standards were averaged for further analysis. The magnetic field data were filtered first between 0.1 and 20 Hz, in order to extract only the transient responses. A subtraction "deviant minus standard" was calculated for visual inspection of the individual data. Assuming the model of an equivalent current dipole (ECD) in a spherical volume conductor a spatio-temporal dipole fit was performed in the latency range of the N1m component elicited by the standard tones. The interval used for the fit (~30 ms) was placed around the local maximum of the global field power derived from the filtered magnetic field data. For each subject two ECDs (one in each hemisphere) were determined, defined by their dipole moment, orientation and spatial coordinates.

The dipole location was determined in a head-based Cartesian coordinate system with the origin at the midpoint of the medio-lateral axis (y-axis), which joined the center points of the entrances to the ear canals (positive toward the left ear). The posterior-anterior axis (x-axis) was oriented from the origin to the nasion (positive toward the nasion), and the inferior-superior axis (z-axis) was perpendicular to the x-y plane (positive toward the vertex). Source locations fulfilling the following anatomical considerations characterizing the human auditory cortex area were included for further analysis: anterior-posterior value (x) within ± 3 cm, medial-lateral value (y - distance from the mid-sagittal plane) greater than 2.5 cm. Additionally, the statistical consideration of goodness of fit larger than 85% derived for source estimations was imposed. Median values of x, y, and z coordinates of the ECDs and of the angles of the dipole orientation were calculated across all blocks, separately for SC and TC. The individual mean values of the source coordinates and orientations of the N1m source were averaged across all subjects in each condition. The average values were used for fix the dipole position and orientation in order to apply the source space projection method [29]. This procedure collapsed the 275 MEG sensor data to two source wave forms representing the activity of the sources in the left and right hemisphere derived for all responses to deviant and to standard tones. These time series reaches a maximum only for a typical dipolar magnetic field pattern of a single current source in an *a priori *specified brain region and therefore this method is spatially sensitive. The source-space projection allows calculation of the grand averages of dipole moment time-series across different subjects and measurement blocks thereby enhancing the signal-to-noise ratio canceling the uncorrelated system noise. The method is maximally sensitive for brain activity from sources at selected origins and orientations. Other neuronal activity from more distant sources or sources having different orientation is combined less optimally and therefore the activity of these sources is reduced in the dipole moment waveforms.

A subtraction of source wave-forms for the *deviants *minus *standards (difference wave form) *resulted in the MMN response. A grand-average across all subjects was calculated for responses to the standard, deviant and subtracted waveform (MMN), and was further analyzed in context of peak amplitudes, latencies and time-courses.

### Statistical analysis

Repeated measurement ANOVAs were conducted for the peak amplitudes and latencies of MMN, N1 and P3a components within the time-window from 100 ms to 350 ms after stimulus onset. Analysis of the auditory evoked responses includes two factors. The first one was "training conditions" consisting of 4 within subjects variables - (i) before training, (ii) immediately after training, (iii) one week after and (iv) one month after training. The second factor "hemispheres", contains two variables, right (RH) and left hemisphere (LH). The contrasts were evaluated by one tailed t-test analysis and post hoc contrasts with the Least Significant Difference test. The comparison between Spectral and Temporal sub-conditions was evaluated by a repeated-measures ANOVA with two factors. The first contains variables of five "conditions" - (i) SC15, (ii) SC25, (iii) TC8, (iv) TC14 and (v) TC18 and the second "hemispheres", contains two variables - RH and LH.

The N1 component was measured from the response to the standard tone; the MMN response was obtained through the subtraction (deviant response - standard response) and the P3a component was measured from the response to the deviant tone. The subject's data, in which the response time course did not contain the investigated components N1 (two subjects) or P3a (one subject), were rejected from the group analysis. The group averaging of the source-waves of the responses to the standard, deviant and subtracted waveforms were estimated in 11 subjects. The 99% confidence intervals for the grand-average *subtracted waveforms *were estimated from non-parametric bootstrap resampling in order to indicate the noise level.

## Results

### Behavioral data

#### Training threshold, Discrimination threshold, Reaction time

**Figure 2:**
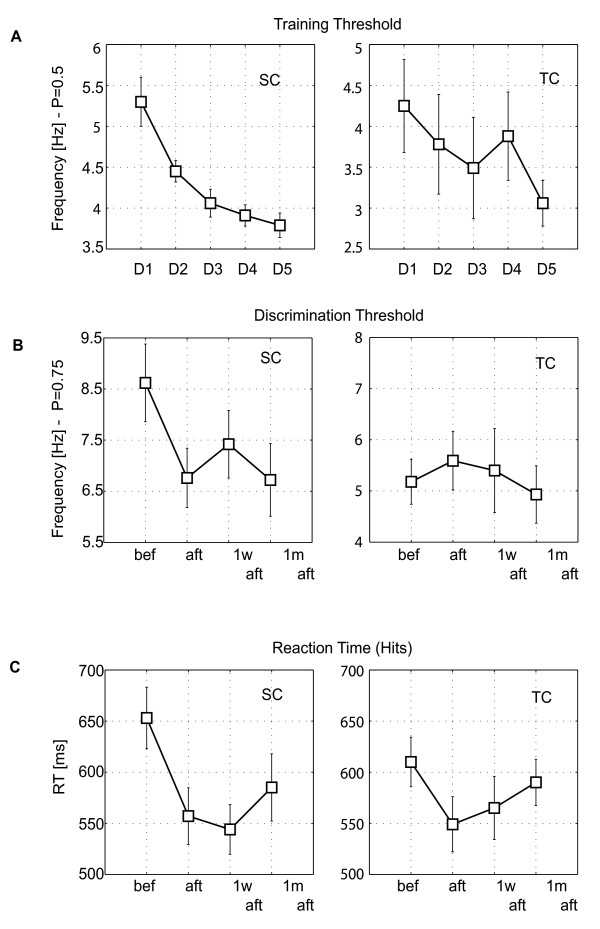
**Behavioral characteristics**. Behavioral data thresholds and reaction time: (A) The training threshold for each day of the training, spectral condition (SC: on the left), temporal condition (TC: on the right); (B) Discrimination threshold in four phases of the training, before training (bef), immediately after the training (aft), one week after the training (1 w aft) and one month after the training (1 m aft), (C) Reaction time for the Hits before, and in the post-training phases. The vertical bars on all graphs represent the standard errors of the means.

The group mean of the behavioral training threshold at the first day of training was 5.3 Hz for SC and 4.2 Hz for TC. At the last day of the training, the thresholds were decreased for both conditions as demonstrated in Figure 2A. The thresholds decreased significantly to 3.7 Hz for SC and to 3.0 Hz for TC (p < 0.01, t-test). In the SC, the tendency of threshold decrease was significant during the training, but in the TC.

We also found a significant decrease of the discrimination threshold in the SC (Figure 2B) decreased significantly after training (*aft*) and one month after training (*1 m aft*, p < 0.05, t-test), but there was no significant decrease in the TC. The reaction time (RT) in SC was significantly shorter directly after training (*aft*), one week after (*1 w aft*), and one month after training (*1 m aft*) as shown in Figure 2C. In TC, however, RT was significantly shorter only immediately after the training (*aft*) compared to before training phase (*bef*), but the RT improvement did not reach significance in the short- (*1 w aft*) and long-term (*1 m aft*) post-training period in comparison to the pre-training test. In general, the behavioral results demonstrate a stronger and stable behavioral training effect in the SC compared to the TC. Significant improvements of the discrimination thresholds and reaction times were found only for the SC immediately after the training and at short and long-term post-training phases.

### Magnetic field data

#### Individual magnetic field responses to changes in carrier frequency (SC condition)

**Figure 3:**
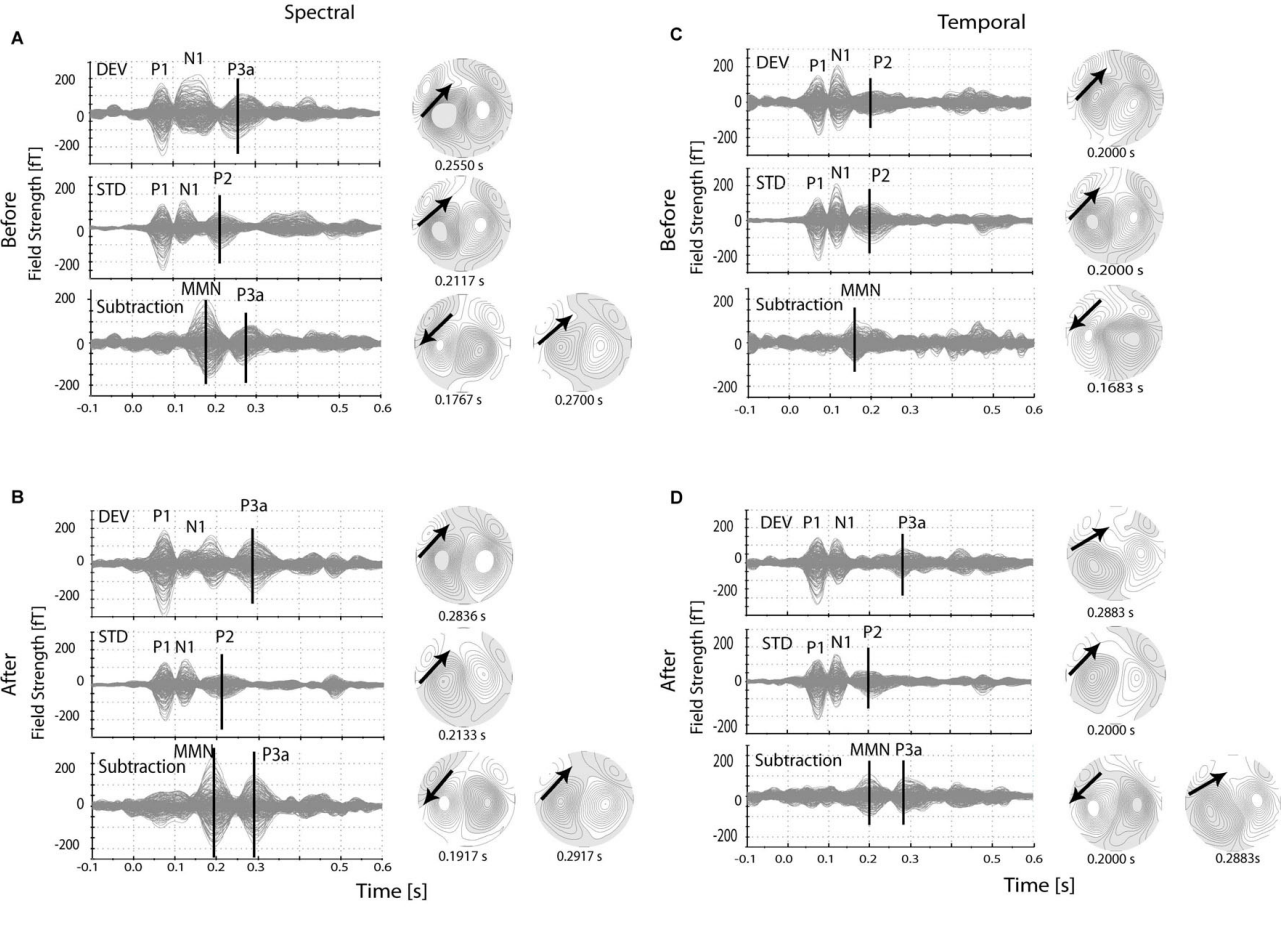
**Single subject magnetic field responses**. Overlay of the MEG channels for to the deviant tone (first row in each plot), standard tone (middle row) and subtracted waveform (bottom row), and the corresponding magnetic field maps for the response components marked with vertical lines on the plots. (A) Magnetic response data in SC before training (Before); (B) Magnetic response data in SC immediately after the training (After); (C) Magnetic response data in TC before training (Before); (D) Magnetic response data in TC immediately after the training (After).

In order to illustrate which transient components were evoked by the change in AM tones (deviant and standard) in an oddball paradigm, individual magnetic field data recorded before and after training were filtered between 2 and 20 Hz and presented in Figure 3. The high-pass filter for this illustration was set to 2 Hz in order to filter out the sustained field from the data, elicited by the long duration of the tones (400 ms). The shows the main components of the response to the deviant tone, to the standard tone, and the subtracted response (deviant - standard) which were used to quantify the auditory discrimination training.

Figure 3A shows the overlayed magnetic channels from individual MEG recording for the spectral sub-condition (SC15) before training. The responses to the deviant and standard tones followed by the subtracted waveform are presented in Figure 3A. A strong dipolar field distribution of each component indicated by a vertical line is demonstrated on the magnetic field maps (on the right). The individual responses before training (Figure 3A) were compared with the corresponding data after training (Figure 3B). The response to the deviant tone (Figure 3A, top panel) consists of three components, P1, N1 and a component with a latency of 255 ms, which we refer to as P3a. The P1 and N1 components can also be seen in the response to the standard tone (Figure 3A, second row), but the N1 shows a smaller amplitude and a reduced half-width compared to the N1 component to the deviant tone. The following positive wave is the P2 with latency around 200 ms. The subtracted response trace (Figure 3A, third row) represents the difference between deviant and standard response. It contains a prominent MMN component (latency of 170 ms) and a small P3a component (latency of 255 ms). The arrow on the magnetic field maps indicates the dipole orientation in the left hemisphere corresponding to the responses marked with bars. Similar field patterns are seen also for the right hemisphere. Figure 3B represents the same responses after training. The training effect, expressed in larger amplitudes, can be seen for both components, MMN and P3a (see Table 1 and Table 2 for significance of source-waves peak amplitude). There is a clear difference between response components to the deviant (N1, P3a) and to the standard tone (N1, P2). The latency difference of the P2 component in the response to the standard tone (≈ 200 ms) and the corresponding positive wave in the response to the deviant (≈ 300 ms) varied between subjects, from 50 ms to 100 ms, and was distinct in the source-wave response curves after the dipole fitting procedure.

**Table 1:** Group average amplitudes and latencies of N1 and MMN components

	Left hemisphere								Right hemisphere							
	**Amplitude [nAm]**				**Latency [ms]**				**Amplitude [nAm]**				**Latency [ms]**			
	
**Response**	***bef*** ***(B)***	***aft*** ***(A)***	***wk aft***	***mo aft***	***bef*** ***(B)***	***aft*** ***(A)***	***wk aft***	***mo aft***	***bef*** ***(B)***	***aft*** ***(A)***	***wk aft***	***mo aft***	***bef*** ***(B)***	***aft*** ***(A)***	***wk aft***	***mo aft***

**N1 (SC15)**	-16.1	-18.1 (mo**)	-16.8	-14.7 (A**)	122	118	117	118	-18.5	-20.7 (mo**)	-19.9	-16.4 (A**)	119	122	114	116

**N1 (SC25)**	-17.5	-16.6	-17.1 (mo**)	-14.1 (B**)	130	123 (B**)	137	122	-21.3	-22.0 (mo***)	-20.2 (mo***)	-16.0 (B**)	127	117 (B**)	116	120

**N1 (TC08)**	-13.2	-16.1 (mo**)	-16.1 (mo**)	-12.8 (A**)	125	127	122	122	-17.0	-18.2 (mo**)	-19.1 (mo***)	-15.9 (A**)	124	120	121	116

**N1 (TC14)**	-17.9	-17.2	-19.1 (mo**)	-17.9 (wk**)	113	116	113	115	-21.5	-21.2	-25.0 (mo**)	-20.3 (wk**)	116	117	113	112
	
	RH>LH **								RH>LH **							

**N1 (TC18)**	-13.9	-16.0	-16.6	-13.8					-17.9	-20.0	-20.0	17.4				
	
	RH>LH (**)								RH>LH (**)							

**MMN(SC15)**	-28.8	-35.2	-33.2	-27.8	199	193	191	190	-31.8	-34.2	-32.6	-29.8	224	194	195	196

**MMN(SC25)**	-32.9	-40.9 (B**)	-34.0	-29.3 (A**)	177	173	174	173	-34.3	-39.9 (B**)	-37.4	-31.5 (A**)	180	177	177	179

**MMN(TC08)**	-17.7	-18.6	-16.2	-18.4	223	214	221	200	-18.0	-19.9	-16.8	-16.7	212	220	206	212

**MMN(TC14)**	-21.9	-21.2	-21.3	-16.5	214	226	204	181	-24.4	-22.9	-22.3	-19.9	207	167	196	184

**MMN(TC18)**	-25.0	-21.8	-23.7	-20.7	194	190	173	177	-26.9	-25.9	-27.9	-24.4	175	198	187	180

(**) P ≤ 0.05

(***) P ≤ 0.001

**Table 2:** Group average amplitudes and latencies of P3a component

	Left hemisphere								Right hemisphere							
	
	Amplitude [nAm]				Latency [ms]				Amplitude [nAm]				Latency [ms]			
**Response**	***bef*** ***(B)***	***aft*** ***(A)***	***wk aft***	***mo aft***	***bef*** ***(B)***	***aft*** ***(A)***	***wk aft***	***mo aft***	***bef*** ***(B)***	***aft*** ***(A)***	***wk aft***	***mo aft***	***bef*** ***(B)***	***aft*** ***(A)***	***wk aft***	***mo aft***

**P3a (SC15)**	-20.1	-12.0 (B**)	-8.7 (B***)	-12.2 (B**)	306	284	286	279	-25.5	-11.1 (B**)	-9.1 (B***)	-10.8 (B**)	283	287	286	274

**P3a (SC25)**	-12.90	-8.56 (B**)	-5.38 (B***) (m**)	-2.02 (B***) (w**)	273	273	274	270	-19.52	-11.69 (B**)	-9.08 (B***) (mo**)	-6.898 (B***) (wk**)	284	270	284	266
	
	LH > RH(**)								LH > RH (**)							

**P3a (TC08)**	-22.3	-18.0	-13.8	-18.4	333	326	319	313	-23.0	-21.3	-16.3	-19.6	318	321	319	315

**P3a (TC14)**	-26.8	-14.2 (B**)	-14.4 (B**)	-14.2 (B**)	308	307	303 (B**)	306	-27.0	-18.3 (B**)	-14.7 (B**)	-16.4 (B**)	330	315	292 (B**)	300

**P3a (TC18)**	-16.4	-11.5	-15.0	-12.4	310	300	294	297	-22.2	-15.2	-12.2	-12.7	293	299	291	291

(**) P ≤ 0.05

(***) P ≤ 0.001

#### Individual magnetic field responses to change in amplitude modulation rate (TC condition)

The magnetic field traces and distribution maps of the responses before and after training in the temporal condition (TC14) are presented in Figure 3CD. In this condition, the deviant tone before the training does not contain a P3a component. The N1 wave was followed by a P2 component, similar to the response components to the standard tone (Figure 3C - first and second row). However, after training a P3a component is visible in the response trace to the deviant tone (latency range between 200 ms and 300 ms after the stimulus onset, Figure 3D - first row). The MMN component after training is followed by a clear P3a component, which is not present in the subtracted response before training.

All individual data in the investigated population showed similar field distributions with respect to the main components described in this example. The MMN response was detectable in all spectral and temporal conditions, but the MMN response amplitudes varied between subjects. There were some conditions and training phases in which the components N1 or P3a were missing in one or two of the subjects.

#### General findings for the MMN source-wave response to spectral (SC-condition) and temporal (TC-condition) changes in an AM tone

**Figure 4:**
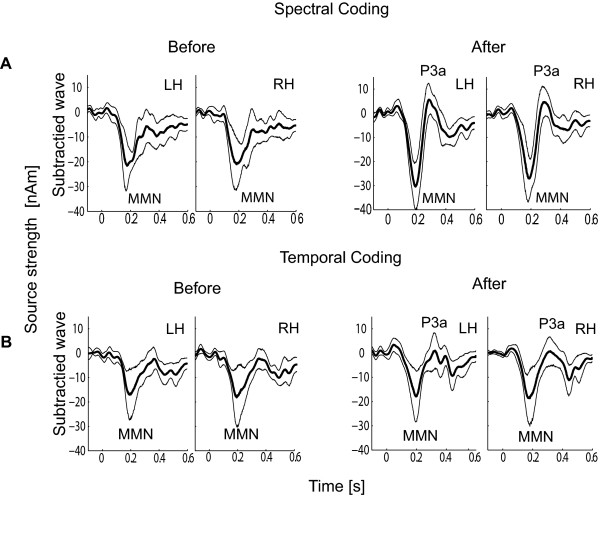
**Group average of the MMN response after dipole fit procedure**. Group average of the subtracted source-waveforms (MMN) across all subjects. (A) MMN response in SC condition before training (Before, on the left side) and immediately after the training (After, on the right side). Thick lines represent the brain response, thin lines the 95% confidence interval after bootstrap procedure across all subjects; (B) MMN responses in TC condition (Before on the left) and after the training (After, on the right). The left and right hemisphere was denoted as LH and RH.

A source projection method was applied to the magnetic field data, after fitting two dipoles for each hemisphere, resulting in a time-course of the dipole moment (one for each hemisphere). This allows group averaging of the responses across all subjects. The difference between deviant and standard response resulting in a subtracted source-waveform was determined for each subject and the corresponding group mean waveform was calculated across all subjects. We found that the smallest spectral difference in the carrier frequency (SC) between AM tones generating a significant MMN was 15 Hz. Correspondingly, the minimal change of the modulation frequency (TC) generating a significant MMN was 8 Hz. Group averaging of subtracted source waveforms in SC15 and TC14 sub-conditions containing the MMN is illustrated in Figure 4. Clear MMN was obtained before and after training from all 11 test-subjects as illustrated in the group results.

The left and right hemispheres are denoted as LH and RH. Thick lines represent the brain response after subtraction. Thin lines represent the 95% confidence interval after a bootstrapping procedure across all subjects. The lower and upper limits of the confidence interval (thin lines) illustrate the significance of the MMN response. If both limit lines are placed below the zero line, the response is significantly different from zero and hence was denoted as significant. The enhancement of the MMN response after training is obvious for the SC15 sub-condition in both hemispheres, but not for the TC14 sub-condition.

Further, the group averaged MMN peak amplitudes listed in Table 1 (across subjects) showed that the maximal MMN amplitude for SC25 was 32.96 nAm in LH and 34.35 nAm in RH. There was no significant difference between amplitudes in both SC sub-conditions SC15 and SC25 as evaluated by a repeated-measures ANOVA. The minimal MMN response amplitude from all three temporal conditions was found for the TC8 sub-condition (17.76 nAm in LH and 18.06 nAm in RH). The MMN amplitude in the TC18 compared to the TC8 sub-condition was significantly larger (p < 0.05). No other comparison within the temporal condition reached significance, nevertheless there is a tendency that an increase of the difference in modulation frequency between standard and deviant increases the MMN amplitudes (see Table 1).

The shortest MMN response latency was observed for the spectral coding sub-condition SC25 (177 ms in LH, 180 ms in RH), the largest latency was measured for the TC8 sub-condition, (223 ms in LH, 212 ms in RH). There were significant differences in the response latencies in SC sub-conditions between SC15 and SC25 (p < 0.02) and in TC sub-conditions between TC8 and TC18 (p < 0.004). In general, larger differences between standard and deviant tones evoked MMN responses with shorter latencies. The MMN latency between spectral and temporal coding conditions was significantly different between SC25 and TC8 (SC25 < TC8, p < 0.01) and between SC25 and TC14 (SC25 < TC14, p < 0.003).

### Training effects

**Figure 5:**
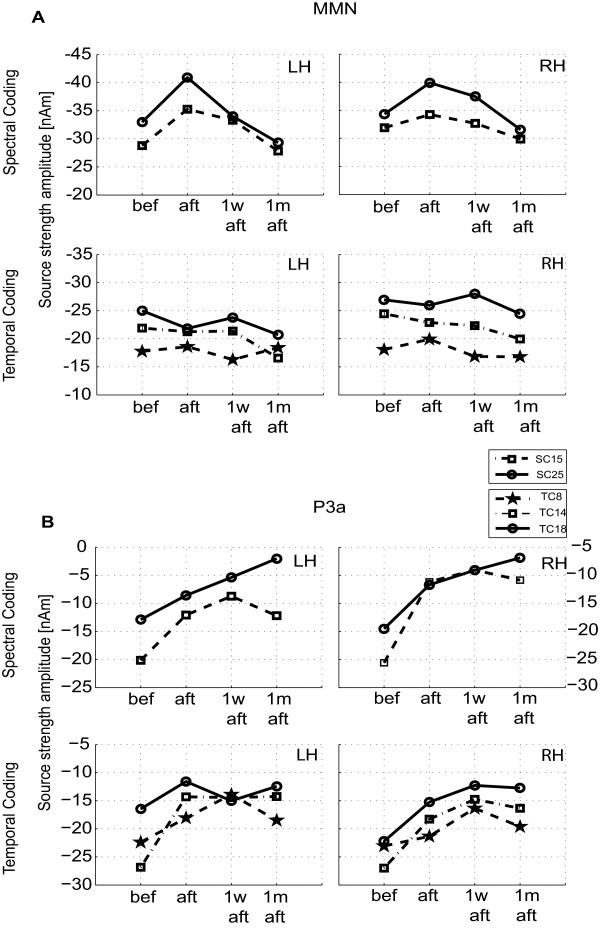
**Peak amplitudes of MMN and P3a response for all conditions**. Group average of the peak amplitudes of the (A) MMN response and (B) P3a response in four training phases: before training (bef), immediately after the training (aft), one week after the training (1 w aft) and one month after the training (1 m aft). The upper and lower rows of the plots represent the SC and TC. Different lines and symbols illustrate the different sub-conditions listed in the boxes.

The source-wave response peak amplitudes and latencies of N1, MMN and P3a were assessed statistically from all training conditions and the results are shown in Figure 5 and as well as in Table 1 and Table 2.

#### Training effects in MMN and P3a for SC conditions

A main effect in the group averaged MMN amplitude after training was observed for SC25, (F(3,30) = 3,6; p = 0.024; Greenhouse-Geisser corrected p = 0.029). Pair-wise comparisons showed that immediately after training and one week after training, the MMN amplitude was enhanced compared to the MMN before and one month after training, where MMN(aft) were larger than MMN(bef) (p = 0.06) and MMN(1 w aft) was larger than MMN(bef), (p = 0.01), Figure 5A (first row). Considerably enhanced amplitudes after training was found for both hemispheres (SC15), however this result failed to reach a statistical significance. The differences in the MMN latencies during training and post training conditions were also non-significant.

The group average of the maximal P3a amplitudes within the SC conditions is depicted in Figure 5B (first row). The P3a amplitude increased after training with a long-lasting effect up to one month after the training. A main training effect of the P3a amplitudes was found in the SC15, (F (3,27) = 7.3; p < 0.001; Greenhouse-Geisser corrected p = 0.004) as well as in SC25 sub-conditions, (F(3,27) = 10.39; p < 0.001; Greenhouse-Geisser corrected p = 0.001). Compared to the P3a amplitude before training the response amplitudes in SC15 sub-condition increased significantly after the training (p = 0.02), one week after the training (p = 0.001) and one month after the training (p = 0.003). In the SC25 sub-condition significant enhancement of the P3a amplitude was found immediately after the training (p = 0.05), one week after the training (p = 0.001) and one month after the training (p = 0.002). The further pair-wise comparison in this sub-condition showed that the P3a amplitude was significantly larger one month after the training, compared with the amplitude immediately after the training (p = 0.04) and compared with the amplitude one week after the training (p = 0.02) (Table 2 and Figure 5B). A lateralization of the P3a response to the left hemisphere was observed for the spectral coding condition with a main effect in the factor "hemisphere" only for SC25 (F(1,9) = 6.64; p = 0.03; Greenhouse-Geisser corrected p = 0.03), Figure 5B - upper graphs).

#### Training effects in MMN and P3a for TC conditions

Figure 5A (lower graphs) shows that the MMN amplitudes in all training phases were not significantly different compared to the baseline measurements. They are higher in RH than in the LH, but the RH lateralization was not statistically significant. No training effects on the amplitudes or latencies were observed for any one of the TC sub-conditions.

The lower graphs in Figure 5B illustrate the increase of the P3a amplitudes after the training for the sub-conditions TC8 and TC14 in the left hemisphere and for the sub-conditions TC8, TC14 and TC18 in the right hemisphere. A main effect of amplitude enhancement after the training was obtained only for TC14 (F(3,24) = 5.96; p = 0.004; Greenhouse-Geisser corrected p = 0.01). This significant P3a amplitude increase in the sub-condition TC14 remained constant in all post training periods (bef - aft, p = 0.018; bef - 1 w aft, p = 0.027; before - 1 m aft, p = 0.004). Main effect for the factor "training" was also observed for the P3a latency ((F(3,24) = 4.05; p = 0.018; Greenhouse-Geisser corrected p = 0.043). The response latency one week after the training was shorter than before training (p = 0.001).

#### Training effects on N1 response wave to the standard tone in SC and TC conditions

Statistical analysis for the SC25 sub-condition showed that there was a main effect in the N1 amplitude for factor "training", (F(3,24) = 3.6; p = 0.029; Greenhouse-Geisser corrected p = 0.06). Immediately after training and oneweek after training the N1 amplitude was significantly higher than the N1 amplitude before training and one month after training (p < 0.01). Similar results were obtained for SC15, but the differences failed to reach a statistical significance. Also in this condition, a N1 latency decrease (p = 0.05) after training was observed as compared to before training.

In the TC condition, a significant enhancement of the N1 amplitudes was obtained for TC8 and TC18 sub-conditions after training (TC8, p = 0.05) and one week after training (TC8, p = 0.01; TC18, p = 0.03). In the TC18 sub-condition a main effect for hemisphere (F(1,8) = 5.51, p = 0.047, Greenhouse-Geisser corrected p = 0.047) was observed; the N1 amplitude in RH was larger than the amplitude in LH (p = 0.047). Additionally, the N1 latency in LH was smaller than in the RH (p = 0.03) with a main effect for the factor "hemisphere" in this condition (F(1,8) = 6.89, p = 0.03, Greenhouse-Geisser corrected p = 0.03).

Similar right lateralization of the N1 was observed also for the TC14 sub-condition. The N1 amplitude was significantly higher in RH, (F(1,8) = 8.51, p = 0.019, Greenhouse-Geisser corrected p = 0.019). See Table 1 for detailed statistical assessment of the various response amplitudes and latencies.

## Discussion

AM tones are characterized by sound envelope fluctuations, which represent temporal features of sounds like speech, music and animal vocalizations. The neuronal mechanisms of AM tone processing involve pitch coding of the carrier frequency, which is tonotopically organized at the cortex, [19,24]. Neuronal encoding of the envelope fluctuation starts in the cochlea and is represented in the cortex by the SSR [20,21,30]. In this study, we investigated auditory cortical change detection responses to small alterations of carrier and modulation frequencies of AM stimuli. We interpreted the MMN and N1 responses as correlates of the cognitive mechanisms for discrimination and change detection in stimulus features, respectively. In addition, the P3a component reported as a neural correlate of an involuntary attention switch towards the deviant or novel sound in an oddball sequence [31-34] was analyzed. Further, the modulation of the response amplitudes and latencies of auditory MMN, N1 and P3a components by behavioral training was statistically estimated during one pre- and three post-training phases (immediately after the training on the 5^th ^training day, one week after the training and one month after the training).

### Generation mechanisms of MMN responses to Spectral and Temporal changes in an AM signal

Our results demonstrated that an MMN response, reflecting more activity in the non-primary auditory cortex, could not only be evoked by frequency (pitch) change but also by change in temporal envelope of an AM tone. Previous studies investigating auditory cortical responses to a frequency change in pure tones revealed that a minimal difference of 15 Hz (3%) perceived as pitch change in pure tones was able to elicit a MMN response [27,35,36]. Similar to these studies, we demonstrated that a 3% difference between the carrier frequencies of two AM tones (spectral change) could elicit a MMN in more than 90% of the investigated subjects. In the temporal condition the smallest difference between modulation frequencies of the deviant (47 Hz) and standard tones (39 Hz) eliciting a significant MMN response was 8 Hz. Both modulation frequencies can be presented in the time-domain as sinusoidal envelopes with periods of 21.2 ms and of 25.6 ms, respectively. The difference between both envelope periods is about 4 ms. Thus, it has been experimentally proven that a 4 ms delay in envelope fluctuations was sufficient to elicit a visible MMN response in more than 50% of the investigated subjects. An enhancement of MMN amplitudes was observed in parallel to the increase of the difference between standard and deviant in the spectral and temporal conditions. Respectively, the MMN response latencies decreased if the difference was larger and the discrimination between standard and deviant was easier. This result was supported by previous studies [35,37] showing that both response parameters (amplitude and latency) correlate with the task difficulty. It is also in line with the results reported by [38], who investigated the stimulus-specific adaptation mechanism (SSA) in the auditory primary cortex of cats. They demonstrated that in an oddball sequence, the response (the number of spikes to a stimulus as a function of its frequency) was stronger to the deviant tone than to the standard tone especially when the frequency difference between standard and deviant tones was larger. The results imply a stronger SSA process to the standard than to the deviant tone, which contributes to the generation of the MMN. An increase of the tone difference between standard and deviant increases the difference between the corresponding neuronal adaptation levels and leads to an increase of the MMN. Therefore, the change detection of smaller differences can be associated with similar SSA to both standard and deviants resulting in a smaller MMN. As established in our results, we suggest that this adaptation mechanism is also involved in detection of differences in an AM tone processed simultaneously at the primary and the non-primary auditory cortex.

The amplitudes and latency deviations between the MMN responses measured for frequency and temporal differences could be explained by the different neuronal mechanisms involved in pitch and envelope neuronal coding. For instance, the spectral cues (carrier frequencies) were coded as pitch in tonotopic cortical maps, whereas the temporal cue of the AM tone (the envelope) was coded as phase locked neuronal activity along the auditory pathway. On the other hand, an AM tone contains spectral and temporal information which is simultaneously analyzed by the auditory system. This could explain certain differences in the results from studies using only pure tones.

Although it is not possible to compare directly the perception of the pitch change with the perception of the envelope change, the results demonstrated almost equal MMN amplitudes in the SCs and in the TC sub-condition (TC18). This similarity between the change detection response to the spectral and temporal difference could be explained in the following way: An AM tone contains two side-band frequency components (Fc ± Fm) in the spectrum around the carrier frequency, producing a roughness in the pitch perception. For larger modulation frequencies, both side-band components are distributed into a wider frequency range. Therefore, the deviant tone corresponding to the largest modulation difference (TC18 sub-condition) could be perceived as a pitch change. This supports better discrimination between standard and deviant and hence leads to an increase of the MMN amplitude, which was equal to the MMN amplitude to the pitch change in the SCs.

### Training effects on the MMN response

We demonstrated that the MMN responses corresponding to frequency change detection (by 15 Hz and 25 Hz) are modulated by discrimination training. The MMN amplitude in the SC25 sub-condition was significantly increased after training in comparison to the MMN amplitude before training and one month after the training. In contrast, there was no change in MMN amplitudes or latencies owing to the training in TC. A significant increase of MMN response amplitude in the SC25 sub-condition is in line with previous results reported by [27] for pure tones. The effect of increased response amplitudes after intensive frequency discrimination training has been explained in previous animal and human studies with the modification of synaptic strength due to practice leading to simultaneous activation of more neurons [1,27,39]. We found smaller cortical response amplitudes in both frequency change and change in modulation rate conditions and smaller amplitude enhancement of the MMN response after training compared to the preliminary study with pure tones of [27]. In the current study, we used longer stimulus duration (400 ms), in order to improve the stimulus perception in change detection of the modulated stimuli. In contrast, the pure tone stimulus duration in the study of [27] was 100 ms. The longer duration might lead to faster adaptation to the stimuli of the neurons generating the cortical responses and thus influencing the measured activity. In a pilot study where we compared MMN responses to both pure and AM tones, we also observed higher response amplitudes to pure tones than to AM tones. These experimental data suggest that the wider spectral content of the AM tone and the longer duration of the stimuli were a possible reason for the observed smaller MMN amplitudes in our study. In our previous study [40] we reported that simultaneously presented AM tones with a modulation rate around 40 Hz interfere and the response frequency specific characteristics show different tuning shapes as compared to those of pure tones. We concluded that this difference might bear on the fact that specific auditory neurons can respond to AM sounds in different frequency bands. Biebel et al. [41] provided evidence for such across-frequency channel interactions by demonstrating that in awake chinchilla the neurons in the inferior colliculus, which were tuned to low-frequency pure tones (e.g., 180 Hz), responded to AM stimuli at carrier frequencies far above the characteristic frequency.

However, the result that in TC the MMN was not modulated by training, neither in the behavioral nor in the MEG data, was surprising. Using simultaneously presented AM stimuli, in the study of [40], it was concluded that a strong widespread interaction between responses occurs only to stimuli containing different carrier frequencies and close situated modulation frequencies. The AM stimuli in our present training condition were presented in tone-pairs with offset to onset inter-stimulus interval of 400 ms. For slow rate sequentially presented stimuli, [42] and [43] reported also widespread neuronal interactions. In SC, the trained stimuli differ only in carrier frequency but not in modulation frequency, whereas in TC the trained stimuli differ only in modulation frequency. Therefore, it could be speculated that the auditory system uses different mechanisms for encoding spectral and temporal information and their interaction. Another study of ours [44] provided further evidence for different mechanisms involved in processing of spectral and temporal stimulus change, demonstrating different latencies of N1 response to spectral and temporal stimulus changes. This is in line with the hypothesis that spectral change could be encoded more quickly, whereas temporal change requires a longer integration time window. In our case, the training in the SC discrimination task results in activation of cortical neurons that respond to the stimuli in the trained carrier frequency range [45,46]. Due to the possible interactions described above, a significant increase in the activity corresponding to the difference was not achieved in the first SC sub-condition (SC15). The reason could be that a small difference in carrier frequency causes overlapping and suppression of activity from adjacent neuronal populations. However, the situation in TC training is different. The spectral content of an AM tone has a peak at the carrier frequency and two side-band frequencies (Fc ± Fm) with half of the power of the carrier frequency (index of modulation equal to 1). Therefore, in the TC training condition the change in modulation frequencies reflects the expansion of the spectral range of the side-band frequencies, whereas the carrier frequency remains identical during training. Hence, the temporal change activated neurons within the tonotopic maps of the same neuronal population (those of the carrier frequency). In this case, the training should affect mainly phase-locked activity due to the envelope fluctuation (i.e., the steady-state activity). Using a precise behavioral training procedure, [16] trained non-musicians to discriminate small increases in the pitch of 2 kHz stimulus, using 40 Hz AM modulated pure tones. They found modulation of the N1, Nc and P2 amplitudes by the training and concluded increasing in synchronous neuronal activity in secondary auditory cortex. In opposite, they reported that the training did not alter the steady-state activity generated in the primary auditory cortex and thus it did not expand the cortical representation for 2 kHz in this area. Therefore, this might suggest that training in discrimination between modulation frequencies (TC) is not sufficient to enhance the neuronal activity of the secondary auditory cortex, resulting in no significant transient response change after the training. The training in the TC condition might introduce neuronal interactions due to modulation channel interference in the primary auditory cortex. Hence, a decrease of this activity in this area could also be suggested, which is in line with findings of [23], who found that a training in discrimination of envelope differences of AM tones suppressed the response from PAC.

### Training effects on P3a components

Recent studies [2,12,46,47] have shown that cortical plasticity due to learning causes task-dependent map reorganization. These plastic changes are investigated while other task-related factors are controlled for, like familiarity with the task or switch of attention. However, a neuronal correlate to the switch of involuntary attention after discrimination training was not described. It is known that the P3a component was often observed as a reaction to stimuli in an oddball paradigm in the presence of a distracting stimulus [32,33] and it has been suggested that it might reflect an attention switch in an unattended auditory task. However, the correlation between behavioral measures (hit rate, reaction time) and neurophysiological properties of P3a (amplitude and latency before and after behavioral training) was not been investigated so far [32].

In our data, the P3a component was measured from the responses to the deviant tone and it was demonstrated that P3a appeared immediately after training in TC and with considerably enhanced amplitude in SCs after training. Interestingly, there was a long lasting training effect on P3a even up to one month after training. This means that sound changes in an oddball sequence could also trigger involuntary attention to a deviant in a simple oddball sequence, which does not involve a distracting stimulus. We observed the P3a component as a reaction to the deviant after exposing the subjects to intensive behavioral training without giving the subjects instructions to pay attention to the stimuli. As reported by [48] rapid discrimination improvement is due to changes in observer's strategies that occur during task familiarization. In our study, the improvement of MMN response was observed only in SC, but the P3a component with enhanced amplitude was clearly discernible after training in both SC and TC. We interpret the enhanced MMN as neuro-plastic change due to the training as an attribute of perceptual learning in a bottom-up process. The P3a component is probably the result of an involuntary attention to the deviant tone due to familiarization to the task as an attribute of rapid learning in a top-down process. The results we obtained here could be associated with the study of [49] who investigated the primary sensory cortex of rats trained to attend to frequency or intensity cues. They suggested that the receptive field plasticity in the cortex is dependent on interaction of top-down and bottom-up processes conferring different stimuli features. Hence, we conclude that discrimination training makes different contributions to the cortical response depending on the different structural features of the stimulation signal (spectral or temporal). Plastic reorganizational changes of the cortical maps due to training are more strongly modulated by the frequency discrimination training, accompanied by an involuntary attention switch to the task dependent stimulus. In contrast, the effect of temporal pattern discrimination training was only due to an involuntary attention switch to the target stimulus that resulted in the generation of the P3a component. It has been demonstrated that musicians could utilize the preattentively encoded neural information more efficiently than non-musicians only when they paid attention to the stimuli, [50]. In other words, even though the brain was trained to recognize very small tone differences in musicians, a voluntary attention process was necessary for better expertise. Therefore, it could be suggested that the discrimination training in our study might cause an involuntary attention switch to the deviant stimuli in TC, but this process has not influenced the discrimination abilities. Consequently, the experimental data demonstrated that the MMN enhancement appearing immediately after the training and remains up to one week after the training only in SC, whereas the P3a component increases immediately after the training but it lasted longer (up to one month) after the training. We could conclude that the improvement of the discriminative abilities after training are not affected by the involuntary attention accompanying the process after the training, but the expertise in encoding neuronal information might be improved only by the task-dependent condition.

## Conclusion

The AM sounds, which are an important part of speech and music, are decoded by the auditory system as sound pitch determined by the carrier frequency and as sound envelope fluctuation determined by the modulation frequency. We investigated neuronal transient responses (N1, MMN) generated mostly in the non-primary auditory cortex to changes in pitch related to the spectral sound content and changes in temporal features of an AM tone regarding envelope fluctuations. Additionally, we demonstrated also, that discrimination training differently affects the cortical responses to carrier frequency changes and to modulation frequencies changes. Therefore, we hypothesized that an additional neuronal mechanism was involved in the processing of differences in AM tones compared to discrimination between pure tones. Finally, after the training the subjects showed an involuntary attention switch to the deviant tone (represented by the P3a-component in the MEG) besides the fact that attention was not prerequisite. This involuntary attention switch, however, does not affect the discrimination ability. In this case, we concluded that the auditory expertise might be influenced only by voluntary attention under task related conditions.

## Abbreviations

AM: amplitude modulation, amplitude modulated; MMN: mismatch negativity; MEG: magnetoencephalography; AER: auditory evoked responses; PAC: primary auditory cortex; SSR: steady-state response; SC: spectral condition; TC: temporal condition; RT: reaction time; LH: left hemisphere; RH: right hemisphere; bef: before training; aft: after training; 1 w aft: one week after training; 1 m aft: one month after training; SSA: stimulus-specific adaptation mechanism; Fc**/**Fm: carrier frequency/modulation frequency; ECD: equivalent current dipole; S-D: standard - deviant; S-S: standard-standard.

## Authors' contributions

RD and CP conceived and designed the experiments, RD and CP interpreted the results, RD carried out data acquisition and analysis. AW, MS and HO contributed to analysis, programming tools and helped in the interpretation of the data, RD wrote the paper, CP, AW, MS and HO revised the manuscript. All authors approved the final manuscript.
